# Effect of right ventricular free wall longitudinal strain on all-cause death in patients with isolated severe tricuspid regurgitation and atrial fibrillation

**DOI:** 10.3389/fcvm.2023.1188005

**Published:** 2023-09-21

**Authors:** Mana Ogawa, Ken Kuwajima, Takafumi Yamane, Hiroko Hasegawa, Nobuichiro Yagi, Takahiro Shiota

**Affiliations:** Smidt Heart Institute, Cedars-Sinai Medical Center, Los Angeles, CA, United States

**Keywords:** atrial fibrillation, tricuspid regurgitation, right ventricular systolic function, right ventricular free wall longitudinal strain, speckle-tracking strain

## Abstract

**Background:**

With the aging population and advanced catheter-based therapy, isolated tricuspid regurgitation (TR) with atrial fibrillation (AF) has gained increased attention; however, data on the prognostic effect of isolated TR with AF are limited because of the small number of patients among those with severe TR. Recently, right ventricular (RV) longitudinal strain by two-dimensional speckle-tracking echocardiography has been reported as an excellent indicator of RV dysfunction in severe TR. However, the prognostic implications of RV longitudinal strain in isolated severe TR associated with AF remain unclear. Therefore, this study aimed to reveal the prognostic value of this index in this population.

**Methods:**

We retrospectively studied patients with severe isolated TR associated with AF in the absence of other etiologies in the Cedars-Sinai Medical Center between April 2015 and March 2018. Baseline clinical and echocardiographic data were studied including RV systolic function evaluated by RV free wall longitudinal strain (FWLS) and conventional parameters. All-cause death was defined as the primary endpoint.

**Results:**

In total, 53 patients (median age, 85 years; female, 60%) with a median follow-up of 433 (60–1567) days were included. Fourteen patients (26%) died, and 66% had right heart failure (RHF) symptoms. By multivariable analysis, reduced RVFWLS was independently associated with all-cause death. Patients with RVFWLS of ≤18% had higher risk of all-cause death adjusted for age (log-rank *P* = 0.030, adjusted hazard ratio 4.00, 95% confidence interval, 1.11–14.4; *P* = 0.034). When patients were stratified into four groups by RHF symptoms and RVFWLS, the group with symptomatic and reduced RVFWLS had the worst outcome.

**Conclusion:**

Reduced RVFWLS was independently associated with all-cause death in patients with isolated severe TR and AF. Our subset classification showed the worst outcome from the combination of RHF symptoms and reduced RVFWLS.

## Introduction

Isolated tricuspid regurgitation (TR) is a phenotype of functional TR without left heart valve disease, left ventricular dysfunction, pulmonary hypertension, and primary TR. Isolated TR associated with atrial fibrillation (AF), which is characterized by right atrial enlargement and tricuspid annular dilatation, has a different mechanism from other functional TR (1). According to the latest guidelines, it is recommended to distinguish isolated TR associated with AF from other types of functional TR (2). Moreover, patients with significant or severe isolated TR associated with AF reportedly had a distinct prognosis compared to patients with other functional TR (3, 4). Isolated TR associated with AF has received much attention because of its growing population with aging (5) and advanced catheter-based therapy, which is a feasible indication for these patients with high surgical risk (6). Previous studies have generally confirmed that significant TR leads to right heart failure (RHF) and poor outcomes (5, 7, 8). However, data as to the prognostic effect on outcome in patients with isolated severe TR associated with AF are limited because of a small population among those with severe TR (9–11).

Recently, right ventricular (RV) longitudinal strain by two-dimensional (2D) speckle-tracking echocardiography has gained attention as an excellent indicator of RV function with feasibility and simplicity in clinical use (12). RV dysfunction is reportedly an essential prognostic determinant in patients with significant TR (13, 14). RV free wall longitudinal strain (FWLS), which measures regional myocardial deformation, has relative angle independence and less load dependence advantages with good reproducibility compared with conventional parameters of RV systolic function, including tricuspid annular plane systolic excursion (TAPSE) and RV fractional area change (FAC) (15, 16). Previous studies have suggested that reduced RVFWLS was associated with poor outcomes in patients with significant functional TR or severe TR (16, 17). In patients with isolated severe TR associated with AF, RV systolic function can be normal using conventional parameters. RVFWLS may detect early RV dysfunction, which could be a critical factor affecting the prognosis of these patients. However, the prognostic effect of RVFWLS on isolated severe TR with AF remains unknown.

Thus, this study aimed to identify the prognostic factors in severe isolated functional TR with AF.

## Materials and methods

### Study population

**Figure 1:**
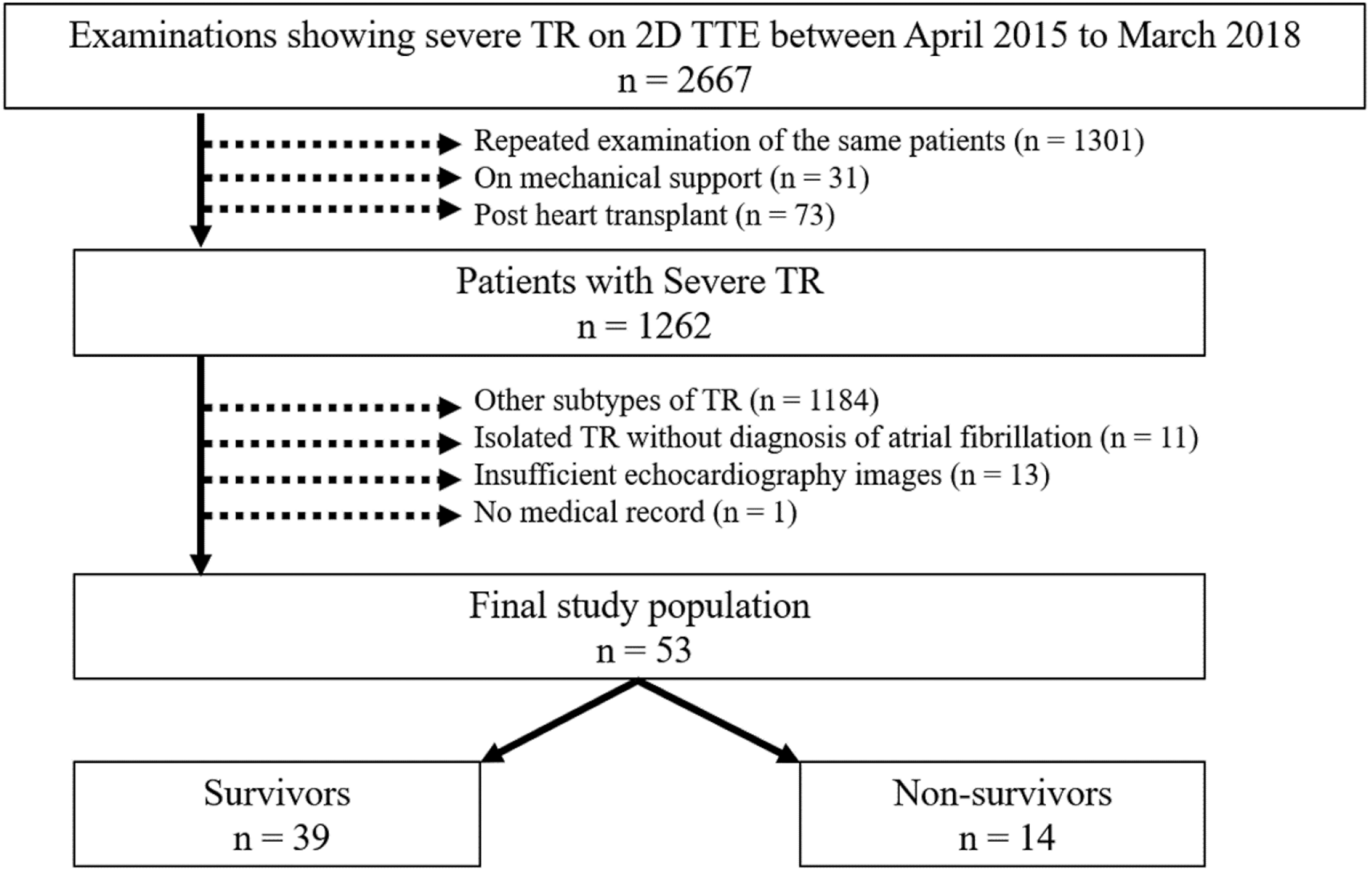
Flowchart of the study population. 2D, two-dimensional; TR, tricuspid regurgitation; TTE, transthoracic echocardiography.

We retrospectively examined 2,667 patients with severe TR from the 2D transthoracic echocardiographic database of the Cedars-Sinai Medical Center (Los Angeles, CA) between April 2015 and March 2018, and patients with isolated TR associated with AF were selected. Isolated TR associated with AF was defined as functional TR with persistent or paroxysmal AF excluding other TR subtypes listed below. AF was diagnosed based on documentation and electrocardiography in the medical records written by the treating physicians according to the guideline (18). The first examination was used when examinations were repeated on the same patient. Patients who had ventilator or assisted circulation devices, previous heart transplant surgery, other TR subtypes, insufficient echocardiography images, and no medical records were excluded. Other TR subtypes included primary TR, functional TR associated with left valvular disease (moderate or more left valvular disease), functional TR associated with left ventricular dysfunction (ejection fraction < 50%), functional TR associated with pulmonary hypertension (systolic pulmonary artery pressure ≥ 50 mm Hg), RV infarction and isolated TR without AF, referring to the classification in a previous study (19). As a result, 53 patients were analyzed for this study (Figure 1).

This study was approved by the Cedars-Sinai Institutional Review Board, and the requirement for informed consent from each patient was waived because of the retrospective nature of this study.

### Clinical data

Data were collected from the electronic medical records and our echocardiographic database. Baseline patient characteristics, including patient backgrounds, comorbidities, and physical examination, were collected at the time of transthoracic echocardiography (TTE) or within three days before or after TTE. With reference to the paper, the symptomatic status of RHF was defined as the presence of edema, ascites, or jugular vein distension, which were evaluated by the treating physicians (20).

### Transthoracic echocardiography

A comprehensive 2D and Doppler TTE was performed by trained echocardiographers using an ultrasound system (S5-1 probe, IE33; Philips, Andover, MA, USA) and experienced cardiologists analyzed TTE images according to the guidelines (21, 22). Integrative assessment of TR severity was performed through a multiparametric approach (including the vena contracta width, color Doppler jet size, and hepatic vein reversal) as recommended by recent guidelines (23). The tricuspid annular diameter was measured on the 4-chamber view at end-diastole. For the tricuspid leaflet tethering height, the vertical distance from the coaptation of the leaflets to the tricuspid annulus line at mid systole was measured. The right atrial (RA) pressure was estimated as 3, 8 or 15 mmHg from the inferior vena cava diameter and its respiratory change (24). The systolic pulmonary artery pressure was calculated based on the TR jet velocity using the modified Bernoulli equation, adding RA pressure (24). The vena contracta width of the TR color jet was measured as the narrowest portion of the jet on the RV-modified apical 4-chamber and the RV inflow parasternal views at mid systole, and the mean value was determined (23). As for right heart evaluation, RV dimensions and RV end-systolic and end-diastolic areas were measured on the RV-focused apical 4-chamber view. RVFAC was calculated according to the guideline (22). For TAPSE, tricuspid annular longitudinal excursion was calculated by the M-mode placing in the line of the lateral tricuspid annular motion as parallel as possible.

### 2D-Echo RV speckle-tracking analysis

**Figure 2:**
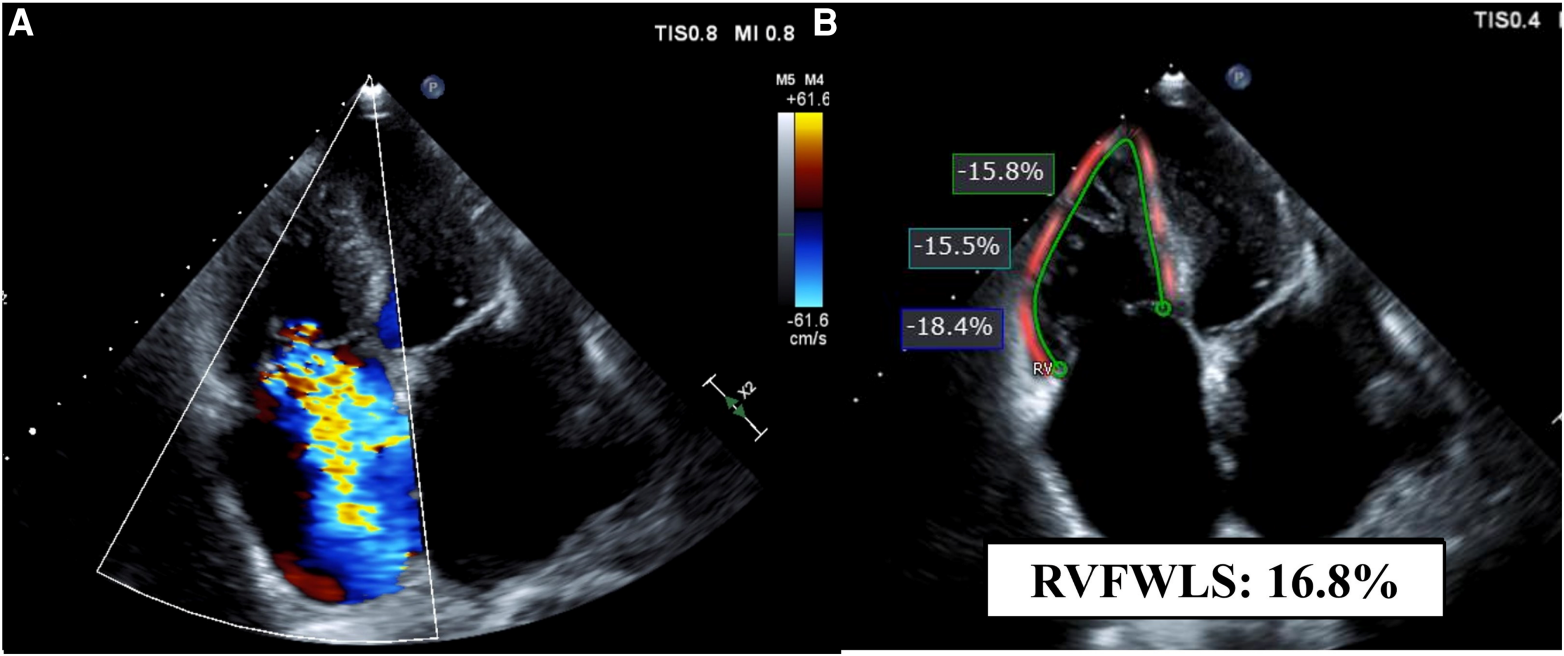
A representative case. Echocardiographic images of a patient with severe tricuspid regurgitation and atrial fibrillation on the RV-focused apical four-chamber view. (**A**) Color Doppler echocardiogram shows severe tricuspid regurgitation. (**B**) Measurement of RVFWLS. FWLS, free wall longitudinal strain; RV, right ventricular.

2D RV longitudinal strain was measured offline using the commercially available software (QLAB, AutoStrain RV; Philips Ultrasound, Inc., Bothell, WA, USA) on the RV-focused apical 4-chamber view by an experienced observer. The RV endocardial border was automatically generated at end-diastole and end-systole with further manual border adjustment if necessary. The software calculated RVFWLS at the RV free wall and RV global longitudinal strain (RVGLS) at the total RV wall, including the ventricular septum as the guideline indicated (Figure 2) (15). In this study, RVFWLS and RVGLS are presented as absolute values. The measurements were averaged from available heart beats (three beats).

### Follow-up data

Follow-up data were collected from the electronic medical records. The clinical outcome in this study was all-cause death. Patients' follow-up was censored at the time of any tricuspid valve (TV) intervention or cardiac transplantation during up to a 7.5-year follow-up.

### Statistical analysis

Continuous variables with normal distributions are presented as mean ± standard deviation and those without as median (interquartile range), which were compared using the unpaired *t*-test or the Mann–Whitney U test as appropriate. Categorical variables were presented as numbers and percentages and were compared using the *χ*2 test or Fisher's exact test. Cox proportional-hazard models were used to determine the independent factors that were associated with all-cause death at follow-up. In the multivariable analysis, covariates showing statistically significant association with all-cause death in the univariable analysis (*P* < 0.05), age or RHF symptoms were included using Cox proportional-hazard models after confirming the absence of strong collinearities. The variables included in the multivariable analysis were selected based on the number of patients and events during the follow-up period. The hazard ratios (HR) and 95% confidence intervals (CI) were obtained. The Kaplan–Meier method and log-rank test were used to describe the event rates of all-cause death during the follow-up period. Receiver operating characteristic (ROC) curve analysis was performed to determine the area under the ROC curves and the optimal cutoff values for the highest sum of sensitivity and specificity using Youden's index. Intraobserver and interobserver variabilities for RVFWLS and RVGLS measurements were obtained by analyzing 15 random cases by the same observer at two time points and two independent blinded observers. The results were analyzed using the intraclass correlation coefficients. *P*-values of <0.05 were considered statistically significant.

All statistical analyses were performed with EZR software (Easy R; Saitama Medical Center, Jichi Medical University, Saitama, Japan), which is a modified version of R (The R Foundation for Statistical Computing, Vienna, Austria) designed to add statistical functions frequently used in biostatistics (25).

## Results

### Patient characteristics

This study included 53 patients with isolated severe TR associated with AF and a median follow-up of 433 (interquartile range, 60–1,567) days. Patients' clinical data according to survival are shown in Table 1. The median patient age was 85 (79–90) years, and 21 (40%) were male. Persistent AF was present in 49 (93%) patients, and 35 (66%) patients had RHF symptoms.

**Table 1:** Clinical characteristics of the total population with isolated severe TR and atrial fibrillation and according to survival.

Variable	Overall	Survivors	Nonsurvivors	*P*-value
	*n* = 53	*n* = 39	*n* = 14	
Age, y	85 (79–90)	86 (79–90)	84 (82–91)	0.52
Male, *n* (%)	21 (40)	14 (36)	7 (50)	0.52
Body surface area, m²	1.75 ± 0.21	1.76 ± 0.22	1.73 ± 0.19	0.68
Hypertension, *n* (%)	42 (79)	29 (74)	13 (93)	0.25
Hypercholesterolemia, *n* (%)	25 (47)	20 (51)	5 (36)	0.37
Diabetes mellitus, *n* (%)	8 (15)	6 (15)	2 (14)	1.00
COPD, *n* (%)	4 (8)	3 (8)	1 (7)	1.00
Coronary artery disease, *n* (%)	12 (23)	8 (21)	4 (29)	0.71
Persistent AF, *n* (%)	49 (93)	36 (92)	13 (93)	1.00
Smoker, *n* (%)	4 (8)	1 (3)	3 (21)	0.052
Liver cirrhosis, *n* (%)	5 (9)	2 (5)	3 (21)	0.11
Hemodialysis, *n* (%)	4 (8)	2 (5)	2 (14)	0.28
Pacemaker/ICD, *n* (%)	16 (30)	14 (36)	2 (14)	0.18
ACE inhibitors or ARBs, *n* (%)	23 (43)	16 (41)	7 (50)	0.75
Beta-blockers, *n* (%)	20 (38)	14 (36)	6 (43)	0.75
Diuretics, *n* (%)	23 (43)	16 (41)	7 (50)	0.75
Aldosterone antagonists, *n* (%)	7 (13)	5 (13)	2 (14)	1.00
Anticoagulants, *n* (%)	25 (47)	20 (51)	5 (36)	0.37
RHF symptoms, *n* (%)	35 (66)	22 (56)	13 (93)	**0.02**
Heart rate, beats/min	71 (60–88)	76 (61–85)	65 (59–93)	0.57
eGFR, ml/min/1.73 m²	32 (23–44)	34 (25–45)	29 (18–36)	0.19
Albumin, g/dl	3.4 ± 0.7	3.4 ± 0.8	3.5 ± 0.5	0.82
AST, U/L	36 ± 18	34 ± 15	38 ± 23	0.65
ALT, U/L	25 (13–34)	25 (14–35)	19 (12–34)	0.40
Total bilirubin, mg/dl	0.8 (0.7–1.4)	0.8 (0.6–1.0)	1.3 (0.7–2.0)	0.16
Hemoglobin, g/dl	11.0 ± 1.7	10.9 ± 1.8	11.3 ± 1.7	0.46
Platelet count, 1,000/UL	177 (133–233)	196 (145–231)	171 (122–245)	0.62
BNP, pg/ml	477 (275–885)	417 (220–587)	922 (376–1,229)	0.052

Data are expressed as mean ± standard deviation, median (interquartile range), or *n* (%). Bold values denote statistical significance at *P* < 0.05.

ACE, angiotensin-converting enzyme; AF, atrial fibrillation; ALT, alanine aminotransferase; ARBs, angiotensin receptor blockers; AST, aspartate aminotransferase; BNP, brain natriuretic peptide; COPD, chronic obstructive pulmonary disease; eGFR, estimated glomerular filtration rate; ICD, implantable cardioverter defibrillator; RHF, right heart failure.

Among 53 patients, 14 (26%) had died. No significant difference in age, sex, and prevalence of comorbidities was found between survivor and nonsurvivor groups. In the nonsurvivor group, the proportion of patients with RHF symptoms was significantly higher than that in the survivor group.

The echocardiographic characteristics according to the outcomes are listed in Table 2. The mean left ventricular ejection fraction was normal and was not significantly different between the groups. Regarding TV and TR parameters including tricuspid annular dimension, tethering height, estimated systolic pulmonary artery pressure, and vena contracta, these parameters were comparable between the two groups. RA volume index, RV diameter, and RV areas also did not differ between the two groups. As for RV systolic function, RVFWLS and RVGLS were significantly lower in the nonsurvivor group than in the survivor group, whereas no significant differences in RVFAC and TAPSE were found between the two groups.

**Table 2:** Echocardiographic characteristics of the total population with isolated severe TR and atrial fibrillation and according to survival.

Variable	Overall	Survivors	Nonsurvivors	*P*-value
	*n* = 53	*n* = 39	*n* = 14	
LV end-diastolic diameter, mm	40 ± 8	40 ± 7	39 ± 9	0.78
LV end-systolic diameter, mm	27 ± 6	27 ± 6	26 ± 6	0.55
LVEF, %	62 ± 7	63 ± 8	61 ± 5	0.50
LA volume index, ml/m²	46 (33–63)	45 (33–58)	50 (44–67)	0.37
RA volume index, ml/m²	70 (54–85)	70 (57–87)	70 (54–83)	0.70
RV basal diameter, mm	49 ± 9	49 ± 10	48 ± 7	0.86
RV mid diameter, mm	39 ± 9	39 ± 9	40 ± 10	0.69
RV longitudinal diameter, mm	67 ± 9	66 ± 10	70 ± 13	0.26
RV end-diastolic area, cm²	26 ± 7	26 ± 7	28 ± 5	0.30
RV end-systolic area, cm²	16 ± 5	16 ± 5	18 ± 4	0.17
RVFAC, %	37 ± 7	38 ± 8	35 ± 6	0.24
TAPSE, mm	17 ± 4	17 ± 4	17 ± 5	0.70
RVFWLS, %	19.4 ± 5.6	20.5 ± 5.4	16.3 ± 5.3	**0.016**
RVGLS %	16.8 ± 4.4	17.7 ± 4.0	14.5 ± 4.5	**0.017**
Tethering height, mm	4.6 ± 3.1	4.4 ± 3.3	5.2 ± 2.4	0.44
Tricuspid annular diameter, mm	42 ± 7	42 ± 7	40 ± 4.5	0.36
Vena contracta, mm	12 (10–14)	12 (10–15)	11 (10–12)	0.33
Estimated SPAP, mmHg	42 (33–46)	43 (35–46)	38 (33–46)	0.59
Maximum IVC diameter, mm	24 ± 7	23 ± 6	24 ± 7	0.55
Minimum IVC diameter, mm	16 ± 6	16 ± 6	16 ± 7	0.60

Data are expressed as mean ± standard deviation and median (interquartile range). Bold values denote statistical significance at *P* < 0.05.

FAC, fractional area change; FWLS, free wall LS; GLS, global LS; IVC, inferior vena cava; LA, left atrium; LS, longitudinal strain; LV, left ventricular; LVEF, LV ejection fraction; RA, right atrial; RV, right ventricular; SPAP, systolic pulmonary artery pressure; TAPSE, tricuspid annular plane systolic excursion.

### Clinical outcomes and prognostic factors for all-cause death

**Figure 3:**
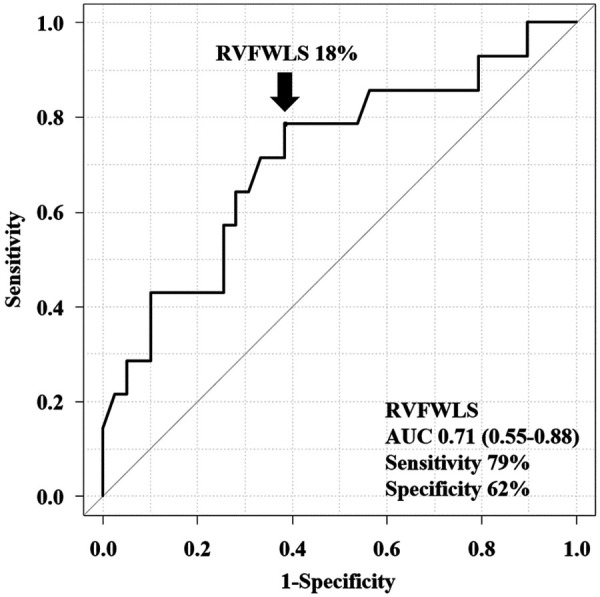
Receiver operating characteristic curve analysis of the ability of RVFWLS to predict all-cause death. RVFWLS 18% was the optimal cutoff value for predicting all-cause death. AUC, area under the curve; other abbreviations as in Figure 2.

**Figure 4:**
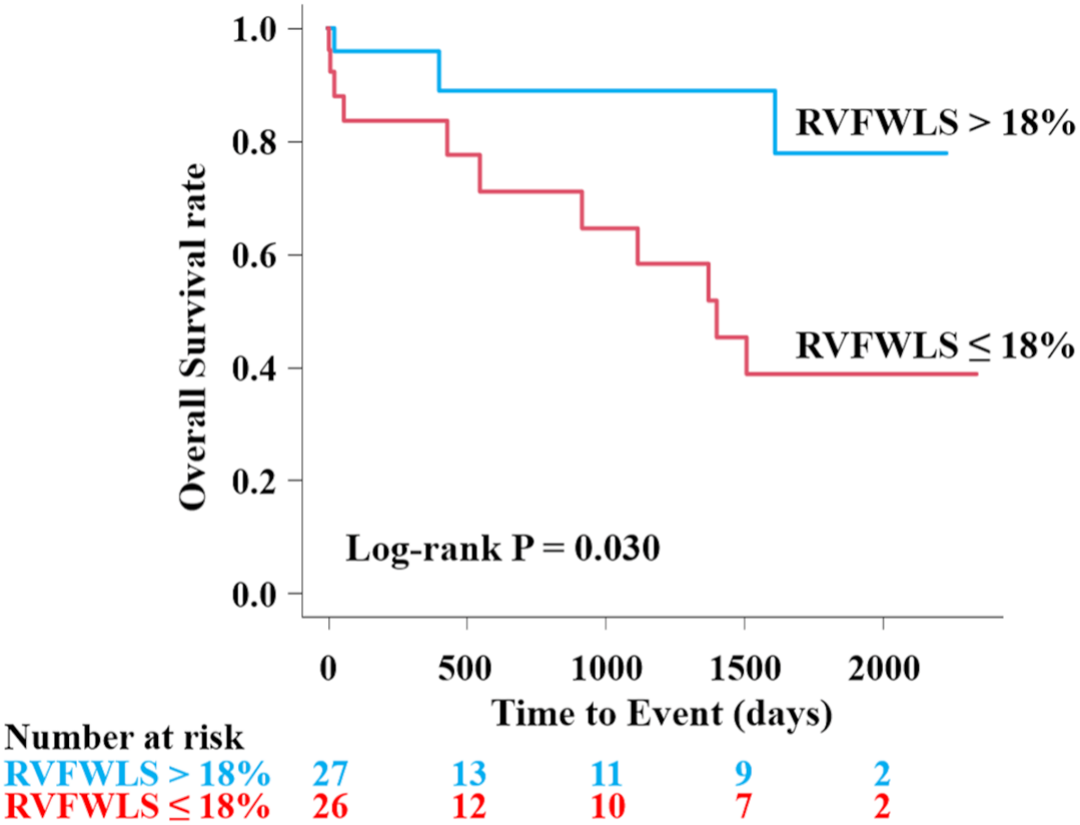
Kaplan–Meier curves according to the presence or absence of RVFWLS ≤18% for predicting all-cause death in patients with severe isolated TR associated with AF. Kaplan–Meier curves showed that RVFWLS of ≤18% was significantly associated with all-cause death in patients with severe isolated TR associated with AF. AF, atrial fibrillation; other abbreviations as in Figures 1, 2.

In the univariable analysis by Cox proportional-hazard regression model, reduced RVFWLS was significantly associated with all-cause death (Table 3). We subsequently drew the ROC curve to determine the optimal cutoff value of RVFWLS for all-cause death. The ROC curve analysis identified that RVFWLS 18% with a sensitivity of 79%, specificity of 62%, and the area under the curves of 0.71 (95% CI 0.55–0.88; Figure 3) is the cutoff value associated with all-cause death. In the multivariate analysis, RVFWLS of ≤18% was a significant predictor of all-cause death after adjusting for age (HR 4.00, 95% CI 1.11–14.4; *P* = 0.034) or after adjusting for RHF symptoms (HR 4.00, 95% CI 1.10–14.5; *P* = 0.035) (Table 3). Kaplan–Meier curves for event-free survival according to the presence or absence of RVFWLS of ≤18% are shown in Figure 4, demonstrating that patients with RVFWLS of ≤18% were at higher risk for all-cause death (log-rank *P* = 0.030).

**Table 3:** Univariable and multivariable Cox regression analysis for all-cause death.

	Univariable analysis		Multivariable analysis Model 1		Multivariable analysis Model 2	
Variable	Hazard ratio (95% CI)	*P*-value	Hazard ratio (95% CI)	*P*-value	Hazard ratio (95% CI)	*P*-value
Age, per 1 year	1.01 (0.94–1.08)	0.79	1.01 (0.94–1.09)	0.81		
Male	1.80 (0.62–5.16)	0.28				
Coronary artery disease	1.44 (0.45–4.60)	0.54				
Persistent AF	3.69 (0.45–30.6)	0.23				
Anticoagulants	0.51 (0.17–1.53)	0.23				
RHF symptoms	6.59 (0.86–50.4)	0.069			6.53 (0.85–50.0)	0.071
Heart rate, per 1 beats/min	1.00 (0.97–1.02)	0.72				
eGFR, per 1 ml/min/1.73 m²	0.96 (0.93–1.00)	0.084				
LVEF, per 1%	0.99 (0.92–1.06)	0.69				
RA volume index, per 1 ml/m²	1.00 (0.98–1.01)	0.55				
RV basal diameter, per 1 mm	1.00 (0.94–1.05)	0.86				
RV mid diameter, per 1 mm	1.02 (0.97–1.08)	0.41				
RV end-diastolic area, per 1 cm²	1.08 (0.99–1.19)	0.085				
RV end-systolic area, per 1 cm²	1.13 (0.99–1.29)	0.062				
RVFAC, per 1%	0.96 (0.89–1.04)	0.31				
TAPSE, per 1 mm	0.92 (0.78–1.10)	0.38				
RVFWLS, per 1% decrease	1.15 (1.02–1.30)	**0** **.** **026**				
RVFWLS ≤18%	4.01 (1.11–14.4)	**0** **.** **034**	4.00 (1.11–14.4)	**0** **.** **034**	4.00 (1.10–14.5)	**0** **.** **035**
RVGLS, per 1% decrease	1.17 (1.02–1.35)	**0** **.** **021**				
Tethering height, per 1 mm	1.10 (0.92–1.31)	0.31				
Tricuspid annular diameter, per 1 mm	0.97 (0.89–1.05)	0.44				
Estimated SPAP, per 1 mmHg	0.98 (0.91–1.05)	0.51				

CI, confidence interval; other abbreviations as in Tables 1, 2.

Bold values denote statistical significance at *P* < 0.05.

### Prognostic assessment based on the combination of RHF symptoms and RVFWLS

**Figure 5:**
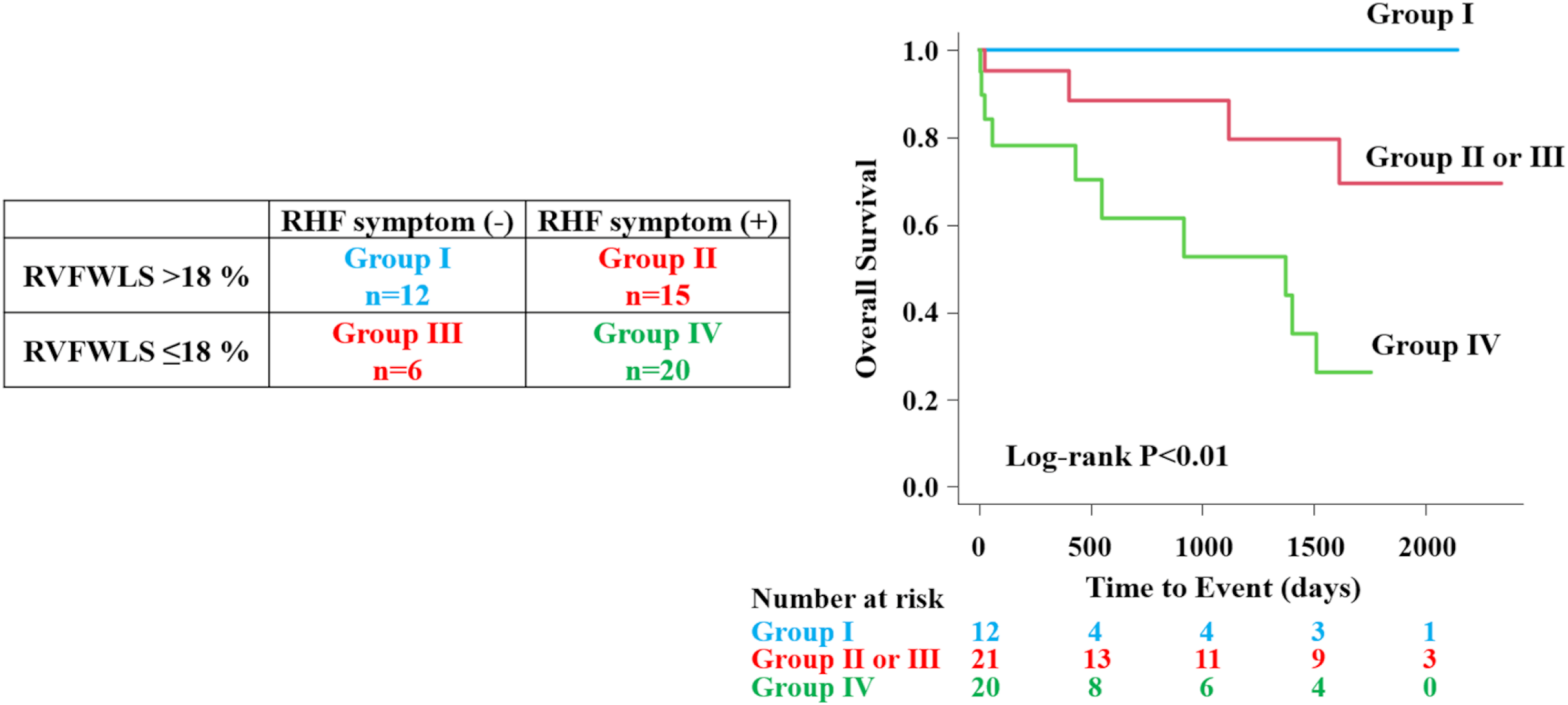
Prognostic assessment according to right heart failure symptoms and RVFWLS. All patients were divided into four groups according to the presence of RHF symptoms and optimal cutoff value of RVFWLS. The patient group that had the combination of RVFWLS ≤ 18% and RHF symptoms had a worst outcome than other groups. RHF, right heart failure; other abbreviations as in Figure 2.

All patients were divided into four groups according to the presence of RHF symptoms and optimal cutoff value (18%) of RVFWLS. The Kaplan–Meier curves showed that group IV patients had the worst prognosis than those in other groups (overall log-rank *P* = 0.005) (Figure 5).

### Reproducibility of RV longitudinal strain

Table 4 showed reproducibility of RV longitudinal strain. These parameters showed good reproducibility for intraobserver and interobserver variability.

**Table 4:** Reproducibility of right ventricular longitudinal strain.

Variable	Intraobserver		Interobserver	
	Variability	ICC (95%CI)	Variability	ICC (95%CI)
RVFWLS, %	1.1 ± 1.0	0.94 (0.82–0.98)	1.8 ± 1.6	0.84 (0.60–0.94)
RVGLS, %	0.92 ± 0.80	0.94 (0.85–0.98)	1.3 ± 1.5	0.86 (0.64–0.95)

ICC, intra-class correlation coefficient; other abbreviations as in Tables 1–3.

### Discussion

Novel findings of this retrospective study are as follows: (1) Nonsurvivors (26%) during up to a 7.5-year follow-up had a lower RVFWLS at the initial evaluation significantly than survivors. (2) Reduced RVFWLS was independently associated with all-cause death. (3) In the subset classification, the combination of RHF defined as RVFWLS of ≤18% and RHF symptoms predicted the worst outcome in patients with isolated severe TR associated with AF.

### RHF symptoms and TR

Severe TR is recognized mainly by RHF symptoms such as jugular venous distension, peripheral edema, dyspnea on exertion, fatigue, ascites, and hepatomegaly (2, 26). However, patients with TR can be asymptomatic for a long time even in advanced RV dysfunction. According to guidelines, RHF symptoms are an important criterion to consider TV surgery in patients with severe TR (2). From our study, 66% of the patients had RHF symptoms, whereas previous studies have shown that 50%–88% of patients with severe isolated TR had RHF symptoms (5, 27). The association between RHF symptoms and prognosis in patients with isolated TR is not fully elucidated yet. Topilsky et al. reported that survival and cardiac event rates were worse in patients with TR and RHF symptoms than those without during the 10-year follow-up period (5). Takahashi et al. reported that cardiac death was higher in patients who were hospitalized for RHF in severe isolated TR associated with AF during a mean 5.9-year follow-up period (11). Research methods with various definitions of isolated TR appear to have caused different results and difficulties in interpretation. Despite the high prevalence of RHF symptoms in nonsurvivors, our study showed that RHF symptoms alone were not associated with poor prognosis, probably because of the small sample size; however, RHF symptoms contributed additively to prognostic stratification in combination with RVFWLS.

### Characteristics of isolated TR associated with AF

Geometric changes of the TV annulus in functional TR associated with AF depended mainly on RA enlargement and the balance between RA and RV remodeling (28). Utsunomiya et al. reported that massive to torrential TR with AF had a larger RV volume index than severe TR with AF (28). However, only a few studies have shown the interaction between prognosis and morphological or functional changes of the right heart in isolated TR associated with AF. In our study, right atrial and TV remodeling, including the RA volume index, tricuspid annular diameter, and tethering height, were not associated with all-cause death, whereas RV dysfunction evaluated by RVFWLS was.

### Effect of RVFWLS on all-cause death

Functional TR imposes chronic volume overload on RV, which causes progressive RV dilatation and dysfunction (17, 19, 29). TAPSE and RVFAC are most frequently reported to assess RV systolic function. However, TAPSE is a one-dimensional measurement and cannot reflect global RV function. On the contrary, RV longitudinal strain, measuring global and regional myocardial deformation, can integrate the information from TAPSE and RVFAC in one measurement with relative angle independence, less load dependence, and high reproducibility. RV longitudinal strain demonstrated higher correlations with RV ejection fraction by cardiac magnetic resonance than TAPSE and RVFAC (30, 31). Previous studies have also reported that RVFWLS aided in detecting RV dysfunction earlier in significant TR than conventional RV parameters (16, 17).

Regarding prognostic factors of isolated severe TR associated with AF, our study showed that reduced RVFWLS was independently associated with all-cause death, whereas other RV function parameters, including RVFAC and TAPSE, were not.

### RVFWLS cutoff value to predict adverse outcomes

A previous study demonstrated that reduced RVFWLS was associated with worse outcomes, when the cutoff value defining RV dysfunction was RVFWLS of <23% in patients with moderate to severe functional TR (16). In our study, only patients with severe TR associated with AF were evaluated. For practical use, we determined the optimal cutoff value of RVFWLS (18%) for all-cause death in the population by drawing the ROC curve. Ancona et al. demonstrated that RVFWLS of <17% predicted the presence of RHF, whereas RVFWLS of <14% was an independent predictor of all-cause death in patients with severe TR (17). Another study using cardiac magnetic resonance to evaluate RV function showed that RVFWLS of ≤16% was an independent predictor of all-cause death in patients with severe functional TR (32). The difference in TR etiology, severity with its definition of TR, and baseline characteristics of the study population may have led to different cutoff values in all these studies. However, our results, in a sense, are consistent with those of previous studies in suggesting that RVFWLS can provide a prognostic value in patients with isolated severe TR associated with AF.

### Classification based on RHF symptoms and RVFWLS

Classifying patients based on RHF symptoms and RV dysfunction can be more practical for clinical management. Dietz et al. reported that higher RHF stages, defined as RV dysfunction based on TAPSE (<17 mm) and clinical signs of RHF, are independently associated with all-cause death at the 5-year follow-up in patients with significant (including moderate) secondary TR, of which 50% had AF (27). They classified the clinical signs of RHF as follows: New York Heart Association functional class ≥II, diuretic use, and/or the presence of peripheral edema. In our study, we did not include moderate or moderate to severe TR to focus on the specific group and used specific RHF signs such as edema, ascites, or jugular vein distension. In patients with isolated severe TR associated with AF, the combination of RHF symptoms and RV dysfunction based on RVFWLS of 18% predicted the poorest outcome.

Preoperative RVFWLS was found to predict prognosis in patients undergoing isolated surgery for severe functional TR with and without AF (33, 34). With the growing attention to the development of transcatheter TV interventions for patients with severe isolated TR in an aging society, standardizing surgical risk stratification is acutely expected. For the optimal timing of surgery and catheter-based intervention in isolated TR associated with AF, sufficient and accumulating evidence of RVFWLS is necessary to avoid irreversible RV dysfunction and end-organ damage.

### Limitations

This study has several limitations. First, this single-center retrospective study had a small sample size, and only patients who were referred for TTE were evaluated. Thus, the statistical power of the analysis may be limited and this analysis might not be generalized to the community. Despite the limited number of patients available for analysis, this study offers valuable insights into the rare patient population with isolated severe TR and AF. Second, systolic pulmonary pressure in the presence of severe TR by Doppler echocardiography could be underestimated, and we could not obtain sufficient data on right heart catheterization; therefore, we may not have excluded patients with pulmonary hypertension completely. Third, we did not evaluate RV systolic function using a three-dimensional echocardiogram, which could measure a more accurate RV volume than 2D evaluation. Fourth, there may be differences in RV longitudinal strain values between different software packages or vendors. The specific cutoff value presented in this study may not be directly applicable to cases using alternative software packages or vendors. Fifth, the AF duration was not evaluated because AF onset could not be identified accurately. Therefore, patients who developed AF after the onset of severe TR could not be excluded. In addition, we did not exclude patients with pacemaker leads. This might fail to exclude patients with iatrogenic TR including pacemaker leads impinging on leaflets. Furthermore, although ascites was evaluated as a RHF symptom, the number of the patients with ascites may be underestimated because ascites was only evaluated from patient records. Finally, due to substantial missing values, we could not include important variables, such as estimated glomerular filtration rate levels, in the multivariable analysis for our study.

## Conclusion

Reduced RVFWLS was independently associated with all-cause death in patients with isolated severe TR and AF. Our subset classification showed the worst outcome from the combination of RHF symptoms and reduced RVFWLS.

## Data Availability

The original contributions presented in the study are included in the article/Supplementary Material, further inquiries can be directed to the corresponding author.
